# Comparison of the sixth and the seventh editions of the UICC classification for intrahepatic cholangiocarcinoma

**DOI:** 10.1186/s40001-018-0329-6

**Published:** 2018-06-01

**Authors:** Benjamin Juntermanns, Gernot Maximilian Kaiser, Lena Orth, Henning Reis, Derar Jaradat, Svenja Sydor, Matthias Buechter, Stefan Kasper, Zoltan Mathé, Georgios Charalambos Sotiropoulos, Hideo Andreas Baba, Ali Canbay, Andreas Paul, Christian Dominik Fingas

**Affiliations:** 1 Department of General, Visceral and Transplantation Surgery, University Hospital Essen, University Duisburg-Essen, Hufelandstrasse 55, 45147 Essen, Germany; 2 Department of General and Visceral Surgery, St. Bernhard-Hospital, Bürgermeister-Schmelzing-Str. 90, 47475 Kamp-Lintfort, Germany; 3 Institute of Pathology, University Hospital Essen, University Duisburg-Essen, Essen, Germany; 4 Department of Gastroenterology and Hepatology, University Hospital Essen, University Duisburg-Essen, Essen, Germany; 5 Department of Medical Oncology, University Hospital Essen, University Duisburg-Essen, Essen, Germany; 6 Department of Transplantation and Surgery, Semmelweis University, Budapest, Hungary

**Keywords:** Cholangiocarcinoma, Intrahepatic cholangiocarcinoma, Liver tumor, Survival, Tumor staging, TNM, UICC

## Abstract

**Background:**

The current seventh edition of the TNM classification for intrahepatic cholangiocarcinoma (ICC) includes tumor number, vascular invasion, lymph node involvement but no longer the tumor size as compared to the sixth edition. The impact of the seventh edition on stage-based prognostic prediction for patients with ICC was evaluated.

**Methods:**

Between 03/2001 and 02/2013, 98 patients with the diagnosis of an ICC were surgically treated at our center. Median survival times were calculated for these patients after separate classification by both sixth and seventh editions.

**Results:**

Median overall survival was increased in patients classified to the lower tumor stages I and II using the seventh as compared to the sixth edition: stage I (54.9 vs. 47.3 months), stage II (19.9 vs. 18.9 months), stage III (17.2 vs. 19.9 months), and stage IV (23.2 vs. 15.3 months), respectively. The seventh edition definition of the T category resulted in an increased median survival regarding the T1 (50.4 vs. 47.3 months) as well as the T2 category (19.9 vs. 15.6 months) and revealed a reduced median survival of patients within the T3 (21.6 vs. 24.8 months) as well as the T4 category (19.9 vs. 27.0 months).

**Conclusions:**

The UICC seventh edition TNM classification for ICC improves separation of patients with intermediate stage tumors as compared to the sixth edition. The prognostic value of the UICC staging system has been improved by the seventh edition.

*Trial registration* The data for this study have been retrospectively registered and the study has been approved by the ethic committee of the medical faculty of the University Hospital of Essen, Germany (license number 15-6353-BO).

## Background

Cholangiocarcinoma is a primary cancer of the bile ducts that arises from malignant transformation of bile duct epithelium [1]. This tumor entity can be classified into intrahepatic cholangiocarcinoma (ICC) and extrahepatic tumors (hilar and distal bile duct) depending on its location within the biliary tree [2]. Ten to fifteen percent of all primary liver cancers are ICCs and these neoplasms are the second most common primary liver cancers following hepatocellular carcinomas. Different incidences have been reported ranging from > 80 per 100,000 population in Thailand to lower incidences in the western world, e.g., Canada with 0.3 per 100,000 population [2–4]. Current studies report that the incidence of ICC is increasing [5, 6]. Comparable to hilar cholangiocarcinoma [7], surgery is the only effective curable treatment up to now [8, 9]. After surgical therapy, the reported 5-year survival rates range between 30 and 35% [9–11]. Therefore, a staging system that exactly separates patients suffering from hilar ICC into prognostic groups is desirable to support patient stratification for treatment with regard to future multimodal perioperative therapeutic strategies. The current seventh edition of the American Joint Committee on Cancer (AJCC) staging manual has introduced a new distinct staging system for ICC based on prognostic factors including tumor number, vascular invasion, lymph node involvement but excluded the tumor size in comparison to the sixth edition [12, 13]. Several studies have focused on the comparison of the sixth and seventh AJCC manual for ICC and found the seventh edition quite suitable for estimation on patients’ prognosis [14, 15]. Comparable to our previous hilar cholangiocarcinoma study [7], the purpose of the present work was to compare the prognostic accuracy of the sixth and the new seventh edition of the AJCC staging systems to predict survival after liver resection for ICC in patients treated at the West German Cancer Center, as one of the largest hepatobiliary centers in Germany.

## Methods

### Patients and specimens

Between 03/2001 and 02/2013, 98 patients with the diagnosis of an ICC were surgically treated at our center. This study was conducted in accordance with the Helsinki Declaration of 1975 and approved by the ethic committee of the medical faculty of the University Hospital of Essen, Germany (license number 15-6353-BO). Routine histopathological workup was conducted for all resected tumors by the Department of Pathology of the University Hospital Essen confirming the diagnosis of an ICC. Our cohort included 42 male (42.86%) and 56 female (57.14%) patients, with an average age of 62.9 (± 11.5) years. All types of resection margins (R0, R1, and R2) and all cases of irresectable disease were included in the study cohort. Follow-up data were prospectively recorded until February 2014.

### Histopathological processing

Surgical specimens were placed in 4% neutral-buffered formalin (12–24 h) prior to histopathological processing, dehydrated, and then cleared using an automated standard procedure (Shandon Pathcentre, Thermo Fisher Scientific Inc., USA) before paraffin embedding in Paraplast (McCormick Scientific, USA). From each paraffin block, 3- to 5-μm sections were cut and Hematoxylin & Eosin slides were prepared adherent to in-house standards. Histopathology reports were available for every case and included macroscopic and microscopic tumor evaluations, in a continuous text summary, and the epicritical report including the TNM (Classification of Malignant Tumors). Data including operative reports and surgical pathology reports of all patients were entered prospectively into a computer database. Cases were stratified according to the sixth and seventh editions of the AJCC/UICC (International Union Against Cancer) TNM classification algorithm for ICC [12, 13]. The sixth and seventh editions of the AJCC/UICC TNM classification algorithm for ICC are compared in Table 1.

**Table 1:** Sixth and seventh editions of the AJCC/UICC TNM classification algorithm for intrahepatic cholangiocarcinoma (ICC)

	Sixth edition		Seventh edition
T1	Solitary tumor without vascular invasion	T1	Solitary tumor without vascular invasion
T2	Solitary tumor with vascular invasion or multiple tumors, none more than 5 cm	T2a	Solitary tumor with vascular invasion
		T2b	Multiple tumors, with or without vascular invasion
T3	Multiple tumors more than 5 cm or tumor involving a major branch of the portal or hepatic veins	T3	Tumor(s) perforating the visceral peritoneum or direct extension to extrahepatic structures
T4	Tumor(s) with direct invasion of adjacent organs other than the gallbladder or with perforation of visceral peritoneum	T4	Tumor with periductal invasion
N0	No regional lymph node metastases	N0	No regional lymph node metastases
N1	Regional lymph node metastases	N1	Regional lymph node metastases
M0	No distant metastases	M0	No distant metastases
M1	Distant metastases	M1	Distant metastases

Stage		Stage	
I	T1 N0 M0	I	T1 N0 M0
II	T2 N0 M0	II	T2 N0 M0
IIIa	T3 N0 M0	III	T3 N0 M0
IIIb	T4 N0 M0		
IIIc	Any T, N1 M0		
IV	Any T, Any N, M1	IVa	T4 N0 M0, Any T N1 M0
		IVb	Any T, Any N, M1

### Statistical analysis

Changes in the distribution of TNM classifications and UICC stages were compared between the sixth and seventh editions, and median survival and survival ranges were calculated independently for each classification. Additionally, 1-, 3-, and 5-year survival rates were calculated. Overall survival was defined as the time from the date of surgery to the date of ICC-specific death or the date of last follow-up. Kaplan–Meier survival plots were generated and comparisons made using the log-rank statistic. All parameters that revealed significant results in univariate Kaplan–Meier analysis were subjected to multivariate Cox proportional hazards regression survival analysis to evaluate the prognostic value of the TNM categories and UICC stages derived from the sixth and seventh editions. Differences of *p* < 0.05 were considered to be statistically significant.

## Results

A total of 98 patients suffering from ICC were surgically treated at our center between 03/2001 and 02/2013. In this retrospective study, we compared the impact of applying either the sixth or seventh editions of UICC tumor staging to stratify median patient survival or predict prognosis in this patient cohort. A summary of differences between the sixth and seventh editions of UICC staging of ICC and the respective TNM categories is presented in Table 1 [12, 13]. We compared the influence of tumor staging using either the sixth or seventh UICC editions on the 1-, 3-, and 5-year survival of patients in this cohort (Table 2).

**Table 2:** Median survival by UICC stage (*n* = 98) using the sixth and seventh editions of the TNM classification

UICC	*N* (%)	Median survival in months (range)	Log-rank test (*p* value)	Cox regression analysis (*p* value)	1-year survival (%)	3-year survival (%)	5-year survival (%)
6th edition			*0.005*	0.053			
I	43 (43.9%)	47.30 (0.30–149.29)			88.4	46.5	23.3
II	8 (8.2%)	18.85 (0.32–97.05)			75.0	12.5	12.5
III	45 (45.9%)	19.90 (0.16–150.11)			64.4	28.9	8.9
IV	2 (2.0%)	15.30 (2.70–27.89)			50.0	0.0	0.0
7th edition			*0.006*	0.082			
I	41 (41.8%)	54.90 (0.30–149.29)			87.8	48.8	24.4
II	26 (26.5%)	19.90 (0.23–97.10)			61.5	19.2	11.5
III	3 (3.1%)	17.20 (5.30–43.20)			66.7	33.3	0.0
IV	28 (28.6%)	23.15 (2.70–150.10)			71.4	28.6	7.1

Survival was calculated using the Kaplan–Meier method and compared using the log-rank test. Cox proportional hazards regression analysis was performed to evaluate the prognostic value of the UICC stage according to the sixth and seventh editions

**Fig. 1:**
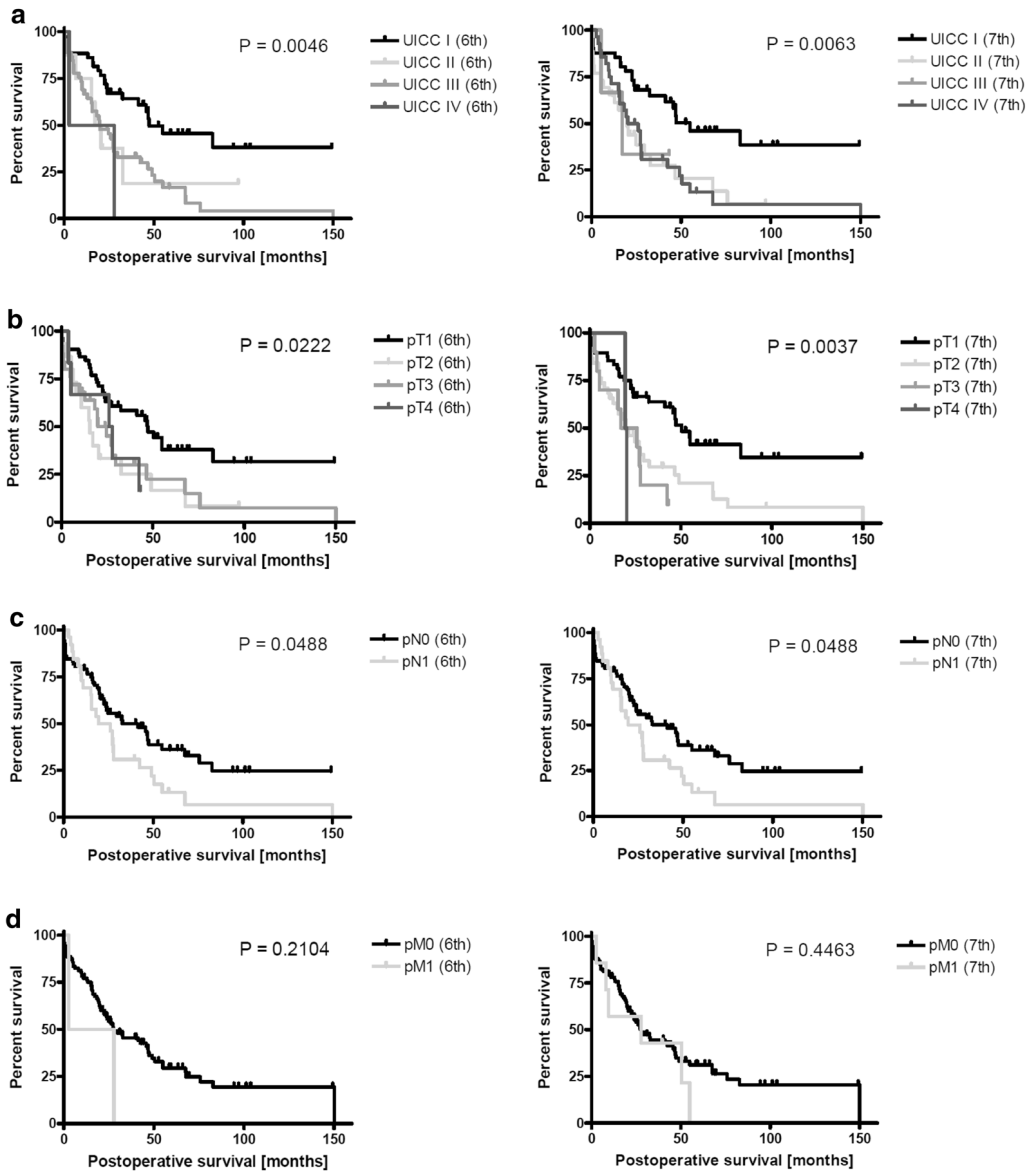
Comparison of survival prediction (*n* = 98) after surgery using the sixth (left) and seventh (right) editions of the UICC tumor classification. Kaplan–Meier analysis was based on tumor stage (**a**) T category, (**b**) N category, (**c**) and M category. (**d**) Significant survival differences (*p* values) were assessed using the log-rank test

The median overall survival for patients was staged according to the sixth or seventh editions and broken down by tumor stage: stage I (47.3 or 54.9 months), stage II (18.85 or 19.9 months), stage III (19.9 or 17.2 months), and stage IV (15.3 or 23.15 months), respectively. Staging according to the seventh edition resulted in an increased median overall survival for patients suffering from ICC in the lower UICC tumor stages I and II. A change in tumor staging occurred in 45 out 98 patients (45.9%) by using the current classification. We also compared the ability of the sixth and seventh editions of the UICC classification to accurately predict patient prognosis based on UICC tumor stage. Kaplan–Meier survival analysis for patients with different UICC tumor stages revealed that the seventh edition more accurately separates these groups (Fig. 1). The prognostic values of the sixth and seventh editions were confirmed by log-rank test and displayed no relevant differences regarding UICC stages (*p* = 0.005 and 0.006, respectively; Table 2).

Regarding the extent of tumor infiltration as reflected by the T (tumor) category, we compared median survival of patients based on either the sixth or seventh edition T category classification (Table 3). The seventh edition definition of the T category resulted in increased median survival in the T1 (50.4 vs. 47.3 months) as well as the T2 category (19.88 vs. 15.6 months) and revealed a reduced median survival in patients within the T3 category (21.62 vs. 24.8 months) and the T4 category (19.86 vs. 26.95 months). The prognostic value of the seventh edition T category staging reached a higher level of significance as compared to the sixth edition T category staging (*p* = 0.004 vs. 0.02 by log-rank test; Table 3). Thus, the distribution shift of patients suffering from multiple tumors from T3 category in T2b category in the seventh classification clearly improved patient stratification particularly for this intermediate tumor stage.

**Table 3:** Median survival by TNM categories (*n* = 98) using the sixth and seventh editions of the TNM classification

UICC	*N* (%)	Median survival in months (range)	Log-rank test (*p* value)	Cox regression analysis (*p* value)	1-year survival (%)	3-year survival (%)	5-year survival (%)
Patients edition			*0.02*	0.27			
T1	52 (53.1%)	47.30 (0.30–149.29)			86.5	44.2	19.2
T2	15 (15.3%)	15.60 (0.23–97.05)			60.0	20.0	13.3
T3	25 (25.5%)	24.80 (0.16–150.11)			64.0	24.0	12.0
T4	6 (6.1%)	26.95 (3.84–43.17)			66.7	33.3	0.0
7th edition			*0.004*	0.09			
T1	48 (49.0%)	50.40 (0.30–149.29)			85.4	47.9	20.8
T2	38 (38.8%)	19.88 (0.16–150.11)			63.2	23.7	13.2
T3	10 (10.2%)	21.62 (2.69–43.17)			70.0	20.0	0.0
T4	2 (2.0%)	19.86 (19.38–20.34)			100.0	0.0	0.0
6th edition			*0.05*	0.74			
N0	72 (73.5%)	41.26 (0.16–149.29)			77.8	36.1	18.1
N1	26 (26.5%)	22.70 (2.69–150.11)			69.2	30.8	7.7
7th edition			*0.05*	0.74			
N0	72 (73.5%)	41.26 (0.16–149.29)			77.8	36.1	18.1
N1	26 (26.5%)	22.70 (2.69–150.11)			69.2	30.8	7.7
6th edition			0.21	N/a			
M0	96 (98.0%)	27.79 (0.16–150.11)			76.0	35.4	15.6
M1	2 (2.0%)	15.29 (2.69–27.89)			50.0	0.0	0.0
7th edition			0.45	N/a			
M0	91 (92.9%)	27.79 (0.16–150.11)			76.9	34.1	16.5
M1	7 (7.1%)	27.89 (2.69–54.97)			57.1	42.9	0.0

Survival was calculated using the Kaplan–Meier method and compared using the log-rank test. Cox proportional hazards regression analysis was performed to evaluate the prognostic value of the TNM categories according to the sixth and seventh editions

Lymph nodes positive for cancer can either be defined as regional spreading of the tumor or as metastases. The definition of regional lymph nodes has been modified within the N (lymph nodes) and M (metastases) categories of the seventh edition of the UICC classification [13]. The overall impact of a positive lymph node on the extent of the disease is emphasized by the seventh edition, such that involvement of any lymph node results in tumor stage IV which has been a tumor stage III in the sixth edition. Applying the seventh edition resulted in reclassification of five patients as M1 based on histological positive lymph node and, therefore, a reclassification from stage IIIc into stage IVa in the current edition (Table 4). Median survival for patients classified for the N category using the sixth and seventh editions was also compared (Table 3). Median survival of patients classified N0 did not change (41.26 months) and also remained the same for N1 (22.7 months). Log-rank test confirmed significance for both editions (*p* = 0.05) regarding the N status.

**Table 4:** Patients with lymph node metastasis upstaged from N1 in the sixth edition to M1 in the seventh edition of the UICC TNM classification

Patients with positive lymph nodes	Location of lymph node infiltration	6th TNM edition	7th TNM edition
Female, 68 years	Celiac artery	N1	M1
Female, 55 years	Portocaval	N1	M1
Female, 47 years	Celiac artery	N1	M1
Female, 64 years	Celiac artery, portocaval	N1	M1
Female, 76 years	Caval	N1	M1

Using the sixth or seventh edition descriptions of the M category also did not affect median survival of patients when staged M0 (27.79 months). Due to the upgrade of five patients to M1 because of distal lymph node metastases (M1), survival in the M1 stage of the seventh classification is prolonged as compared to the sixth classification (27.89 vs. 15.29 months, no significance; Tables 3, 4).

## Discussion

In this single-institution study, we found that the seventh edition of the UICC TNM classification for ICC slightly more accurately stratifies patients suffering from this neoplasm. Particularly, the guidelines for the current seventh edition AJCC staging manual has introduced a new distinct staging system for ICC based upon prognostic factors including tumor number, vascular invasion, lymph node involvement but no longer the tumor size as opposed to the sixth edition [12, 13]. This redefinition enables a more appropriate patient stratification in our cohort. Another novelty is the new definition of distant lymph node metastases resulting in several tumor stage upgrades in our cohort with a change of median survival regarding UICC tumor stage IV.

Tumor staging according to AJCC/UICC seventh edition was accompanied by an increased survival in tumor stages I and II allowing a more appropriate prognosis stratification. The reason for this was an altered classification of 45 out of 98 patients using the current classification. Thus, a total of 67 (seventh edition) instead of 51 (sixth edition) patients is classified to the lower tumor stages I and II in this study. This finding is consistent with a multicenter study conducted by Ribero et al. [14]. This group also reported that the majority of their patients displayed a T1 or a T2 tumor stage and an UICC/AJCC stage I or II. In our opinion, this new distribution allows a better stratification within our cohort and, therefore, a better selection for adjuvant therapy strategies.

We also found several changes regarding the T status applying the seventh edition. In our cohort, the new T categories enabled a more appropriate patient stratification, especially for T1 and T2. For T2, this fact is due to the loss of tumor size evaluation causing a downstaging for multiple tumors from T3 to T2b. Nathan et al. [15] could demonstrate that the tumor size does not correlate with any additional prognostic value by analyzing the survival data of 598 patients following resection for ICC. Thus, they concluded that the sixth edition AJCC/UICC T classification failed to stratify T2 and T3 cohorts into two distinct prognostic groups. Our data support this finding as the prognostic value of the seventh edition T category staging reached a higher level of significance as compared to the sixth edition T category staging (*p* = 0.004 vs. 0.02). Another novelty in the seventh AJCC/UICC staging system is the introduction of a separate classification of the T4 category “periductal tumor infiltration.” In this study, only two patients were found to show periductal infiltration. Both patients also displayed positive lymph node metastasis and were therefore staged as AJCC/UICC IVa. Thus, the prognostic significance of this new T4 category remains uncertain in our cohort and needs to be investigated more specifically in upcoming studies with larger patient numbers.

The seventh edition also re-evaluates the presence of regional lymph node involvement regarding the N and M classifications emphasizing the importance lymph nodes infiltrated with cancer for UICC tumor staging. This resulted in the reclassification of patients with lymph node metastases into UICC stage IV by the seventh edition (formerly stage III in the sixth edition). Additionally, distal lymph nodes were reclassified in the current classification manual [13]. This novelty affected a total of five patients in our cohort categorized as stage III according to the sixth edition of the TNM classification. Due to a celiac or mesenteric lymph node infiltration (N1), the tumors were upstaged to metastatic (M1, stage IV) disease according to the seventh edition (Table 4). Similar to our previous study of perihilar cholangiocarcinoma [7], this novelty did not significantly affect patient survival comparing both groups (Table 3). Interestingly, the new M staging in our collective caused a prolonged survival in the M1 group. This finding raises the question, if the upgrade from N1 to M1 really mirrors an altered prognosis as intended by the AJCC/UICC. Nevertheless, Farges et al. [16] recommended that a routine lymphadenectomy at the time of surgery for ICC should become the standard of care, which again emphasizes the importance of lymph node involvement.

## Conclusion

In conclusion, based on this study of 98 patients treated for ICC at a single institution, the categorization of UICC tumor stages by the seventh UICC edition enables a better patient stratification than the sixth edition. The current edition emphasizes the importance of lymph node involvement and periductal infiltration instead of tumor size. Thus, the seventh edition more appropriate separates intermediate tumor stages as reflected by the median patient survival for this cohort and confers a higher prognostic value to the tumor stage. This should facilitate the stratification of patients diagnosed with ICC into different risk groups resulting in better customized multimodal perioperative treatment strategies. Our data show that particularly the T categories of the seventh edition enable a more appropriate prediction of patient survival than the corresponding categories from the sixth edition. A considerable number of patients were staged differently by the seventh edition in the present study. This fact should be considered when comparing previous studies employing the sixth edition with new data from ICC patients. Finally, the T4 category (periductal infiltration) and the reclassification of distant lymph nodes need to be re-evaluated in upcoming studies with larger patient numbers.

## Abbreviations

ICC: intrahepatic cholangiocarcinoma
AJCC: American Joint Committee on Cancer
TNM: Classification of Malignant Tumors
UICC: International Union Against Cancer
T: tumor
N: lymph node
M: metastases
